# Ohio State Veterinary Medical Association

**Published:** 1894-03

**Authors:** 


### OHIO STATE VETERINARY MEDICAL ASSOCIATION.

The annual meeting of the Ohio State Veterinary Medical Association was held at Columbus, Ohio, on January 9th and 10th, 1894.

*First Day's Session.*

Dr. J. D. Fair, the Vice-President, in the chair. The attendance of members was good, and augmented by several visitors, Dr. T. C. McQuade, Canton, O., Dr. W. C. Fair, Cleveland, O., and Dr. Styer, of London.

The election of officers resulted in the unanimous choice of:

*President*—Dr. J. D. Fair, Berlin, O.
*Vice-President*—Dr. Neil Jones, Cincinnati, O.
*Second Vice-President*--Dr. F. E. Anderson, Findley, O.
*Secretary*—Dr. W. H. Gribble, Washington C. H., O.
*Treasurer*—Dr. T. B. Hillock, Columbus, O.

*Dr. Collon* presented the following amendment to the by-laws, which had been duly filed at a previous meeting.

Strike out from the Code of Ethics, sections 5 and 6.

SECTION 5. In advertising, the Veterinary Surgeon shall confine himself to his business address. Advertising specific medicines, specific plans of treatment, advertising through the medium of posters, illustrated bills, newspaper puffs, etc., will not be tolerated by this Society.

SECTION 6. Secret Medicines. Any members who shall advertise or otherwise offer to the public any medicines, the composition of which he refuses to disclose, or who proposes to cure diseases by any such secret medicines, he shall be denounced as an unworthy member, and shall be expelled from the Association.

After considerable discussion the amendment was laid on the table.

*Dr. Anderson* reported the removal of a splint in three days by some application.

An Auditing Committee, Drs. Jones, Howe and Bretz, was appointed to examine the books of the Association. Adjourned.

*Second Day's Session.*

Dr. J. D. Fair in the chair.

The Auditing Committee reported:

"We, your committee appointed to audit the books of this Association, beg leave to report that we find the books well kept and the association treasury in a flourishing condition, there being at the beginning of this session in the hands of the Treasurer, $305.54."

NEIL JONES,
W. R. HOWE,
S. F. BRETZ,
*Committee.*

Letters were read stating that the President, Dr. Wright, was ill, and that Dr. George Butler was disabled by severe injuries.

Dr. Hillock was called to the chair while Dr. Fair presented a paper of interesting cases of fracture, melanosis, purpura and spinal meningitis.

*Dr. Neil Jones* stated that only recently his first success in fractures had altered his opinion of the good results to be obtained by treatment. He has lately had a compound comminuted fracture in a cow, and a compound fracture of the radius of a horse, make a favorable recovery.

On discussion of the treatment of purpura, the majority favored the use of strychnine and the iodide of iron.

*Dr. Neil Jones* stated that uncalled for and ignorant criticism of the Ohio Veterinary College had been made at a recent meeting of the New York State Veterinary Medical Society. (See Dr. Jones' letter, page 218. Ed.)

*Dr. Gribble* presented a paper on "Life and Death."

*Dr. Howe* reported recovery from a case of rupture of the ligaments in the neck by the use of splints. Tetanus followed sores caused by the splints, but the animal recovered from this.

Millersburgh was selected as the next place of meeting in September, 1894, during the exhibition of the Holmes County Fair.

---

### VETERINARY MEDICAL ASSOCIATION OF NEW JERSEY.

Tenth Annual Meeting on Thursday, April 12th, 1894, at State Street Hotel, Trenton, New Jersey.

Full attendance of members expected.

Delegates from other Veterinary Associations invited.

S. LOCKWOOD, *Secretary.*
Woodbridge, N. J.

